# Imaging Voltage Globally and in Isofrequency Lamina in Slices of Mouse Ventral Cochlear Nucleus

**DOI:** 10.1523/ENEURO.0465-22.2023

**Published:** 2023-03-02

**Authors:** Yihe Ma, Wen-Chi Shu, Lin Lin, Xiao-Jie Cao, Donata Oertel, Philip H. Smith, Meyer B. Jackson

**Affiliations:** Department of Neuroscience, University of Wisconsin School of Medicine and Public Health, Madison, WI 53705

**Keywords:** auditory nerve, c-fos, cochlear nuclei, genetically-encoded voltage sensors, tonotopic organization, voltage imaging

## Abstract

The cochlear nuclei (CNs) receive sensory information from the ear and perform fundamental computations before relaying this information to higher processing centers. These computations are performed by distinct types of neurons interconnected in circuits dedicated to the specialized roles of the auditory system. In the present study, we explored the use of voltage imaging to investigate CN circuitry. We tested two approaches based on fundamentally different voltage sensing technologies. Using a voltage-sensitive dye we recorded glutamate receptor-independent signals arising predominantly from axons. The mean conduction velocity of these fibers of 0.27 m/s was rapid but in range with other unmyelinated axons. We then used a genetically-encoded hybrid voltage sensor (hVOS) to image voltage from a specific population of neurons. Probe expression was controlled using Cre recombinase linked to *c-fos* activation. This activity-induced gene enabled targeting of neurons that are activated when a mouse hears a pure 15-kHz tone. In CN slices from these animals auditory nerve fiber stimulation elicited a glutamate receptor-dependent depolarization in hVOS probe-labeled neurons. These cells resided within a band corresponding to an isofrequency lamina, and responded with a high degree of synchrony. In contrast to the axonal origin of voltage-sensitive dye signals, hVOS signals represent predominantly postsynaptic responses. The introduction of voltage imaging to the CN creates the opportunity to investigate auditory processing circuitry in populations of neurons targeted on the basis of their genetic identity and their roles in sensory processing.

## Significance Statement

The cochlear nucleus (CN) uses dedicated circuitry to process and interpret information from the ear. This circuitry is organized tonotopically into laminae, each containing cells with an optimal sensitivity to a specific sound frequency. By targeting a genetically-encoded hybrid voltage sensor (hVOS) to identify neurons activated during the presentation of sound, the properties and function of these neurons become accessible to study in slices of mouse ventral cochlear nucleus (VCN). Imaging hVOS signals in these slices recapitulated the tonotopic organization. Imaging with a voltage sensitive dye provided a contrasting global view of signals arising predominantly from unmyelinated axons creating a potential method for studying Type II auditory nerve or DCN parallel fibers.

## Introduction

The cochlear nucleus (CN) receives the entire synaptic output of the auditory nerve (AN). This bundle of fibers enters the CN at the nerve root area, and divides the ventral cochlear nucleus (VCN) into anteroventral (aVCN) and posteroventral (pVCN) subdivisions. Approximately 95% of the AN fibers are designated Type I: they are myelinated and arise from spiral ganglion cells whose peripheral processes receive their input from a single inner hair cell (Kiang et al., 1982). The remainder are designated Type II; they are unmyelinated and innervate multiple outer hair cells (Kiang et al., 1982). Almost every Type I and II AN axon bifurcates at the nerve root area and sends an ascending branch rostrally into the aVCN and a descending branch caudally into the pVCN (Brown et al., 1988; Liberman, 1991, 1993). Most of the descending branches then typically curve rostrally to innervate the dorsal cochlear nucleus (DCN) as well. The projection and distribution of both ascending and descending branches of Type I AN axons are organized according to their best or characteristic frequency (CF), the frequency of sound with the lowest spike threshold (Liberman, 1991, 1993; Tsuji and Liberman, 1997). Low CF AN fibers terminate more ventrally and higher CF fibers more dorsally. Each AN fiber has collateral branches that are usually short and make both en passant and en terminaux synapses on CN cells (Fekete et al., 1984; Rouiller et al., 1986; Ryugo and Rouiller, 1988; Liberman, 1991, 1993). The outcome of the Type I fiber tonotopic projection is that CN neurons receiving this input are likewise organized into narrow isofrequency laminae in a tonotopic fashion with cells in each lamina in each CN subdivision showing a consistent CF (Osen, 1970; Kiang et al., 1975; Bourk et al., 1981; Ryan et al., 1982; Willott et al., 1982; Luo et al., 2009).

Most of the electrical response data from CN neurons has been collected using single-cell recording techniques, both *in vivo* and *in vitro*. These kinds of experiments require a considerable amount of labor and many experimental animals to collect enough data for meaningful inferences. Additionally, if one is interested in cells with a particular feature, for example cells in a given isofrequency lamina with the same CF, it is even more difficult. Furthermore, with *in vitro* slice preparations one can only approximate the CF of a recorded cell based on its location. In a slice it would be of great advantage to be able to evaluate the simultaneous activity of many cells with the same CF.

Imaging with voltage sensors can address questions about circuit function by detecting electrical activity in large numbers of individual neurons as well as in spatially resolved populations of neurons. Furthermore, the targeting of genetically-encoded voltage sensors enables the imaging of electrical activity from specific cell types with defined functions. Among the many experimental approaches to voltage imaging (Canepari et al., 2015), we introduce two complementary techniques to study dynamic activity of different neuronal populations in slices of mouse VCN. Voltage sensitive dye imaging employs a synthetic probe to label all cells nonselectively. This approach revealed the collective activity of axons. We then imaged activity selectively in neurons previously activated in the mouse when it hears a particular sound frequency. This was accomplished by combining the approach referred to as targeted recombination in active populations (TRAP; Guenthner et al., 2013) with a genetically-encoded hybrid voltage sensor (hVOS; Chanda et al., 2005). Genetically encoded voltage sensors can be targeted to specific cell types (Lou et al., 2016; Bayguinov et al., 2017; Song et al., 2018; Platisa et al., 2022) and hVOS probes have also been used to target specific populations based on prior activity (Bayguinov et al., 2017). The TRAP method links Cre recombinase expression to the early-immediate gene *c-fos,* a gene that is activated in neurons when they are electrically active (Guenthner et al., 2013). This allowed us to target hVOS probe selectively to neurons that had previously been activated by a specific form of auditory stimulation in live animals, in this case a 15-kHz tone. This method permitted subsequent *in vitro* recordings of neural activity from a population of cells within an isofrequency band. The voltage-sensitive dye signals revealed the conduction of action potentials in axons, while the hVOS signals revealed primarily postsynaptic responses of neurons within an isofrequency lamina. The rapid response times of both voltage sensors enabled us to resolve critical dynamic processes that are too rapid to see with calcium sensors (Zhu et al., 2021). In this work, we focus on the pVCN and explore the use of these imaging methods to characterize circuit activity within an isofrequency lamina. In the future, when combined with genetic or other labeling methods that identify specific cell types, these methods could potentially provide powerful approaches for elucidating the circuitry of specific cell types within an isofrequency band under both normal and abnormal (e.g., noise damaged) conditions.

## Materials and Methods

### Animals

The animal protocols used in the present study were approved by the Animal Care and Use Committee of this institution, in accordance with the recommendations in the *Guide for the Care and Use of Laboratory Animals* of the National Institutes of Health.

Mice for voltage-sensitive dye imaging came from a colony of CBA/CaJ mice bred in-house (stock #000654; The Jackson Laboratory). CBA mice are commonly used in auditory electrophysiology studies, and their hearing is similar to that of C57 mice (Walton et al., 1995). When these mice were not available, we purchased outbred ICR mice from Envigo (formerly Harlan Laboratories). Postnatal day (P)16–P22 animals of either sex were used. Auditory brainstem responses were qualitatively similar between strains. Probe expression and imaging signals were qualitatively similar between sexes.

Mice for hVOS imaging and immunohistochemistry staining were bred in the Biotron Laboratory of UW-Madison and our in-house facility. Fos-CreER (TRAP) mice (Guenthner et al., 2013) were purchased from the Jackson Laboratory (B6.129(Cg)- Fos<tm1.1(cre/ERT2)Luo>/J, stock #021882). Our Cre reporter Ai35-hVOS mice (Bayguinov et al., 2017) are available from the Jackson Laboratory (stock #031102). FosCreER+/− males were mated with hVOS^+/+^ females to obtain litters with 50% harboring both genes (referred to here as TRAP::hVOS mice). Genotyping was performed after P14 to confirm the presence of the hVOS and Cre genes. No difference in weight, appearance, and behavior was noticed between FosCreER+/− mice and their FosCreER−/− littermates.

### Auditory stimulation

**Figure 1.**
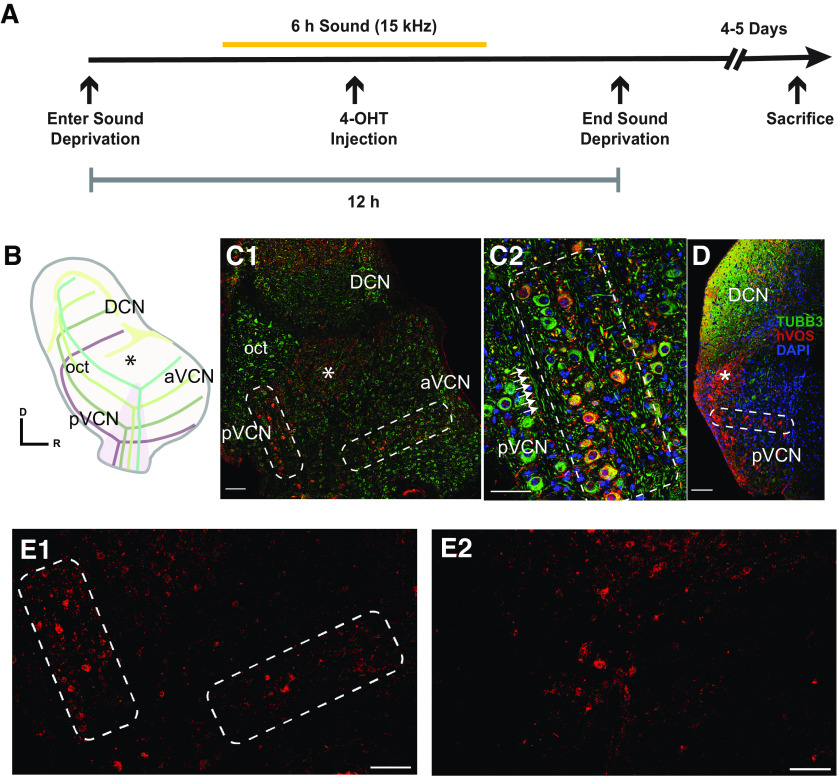
Labeling of neurons activated by pure tone stimulation in the CN of TRAP::hVOS animals. ***A***, Auditory stimulation paradigm (see Materials and Methods): TRAP::hVOS mice in a soundproof box experienced 3 h of silence followed by exposure to a 6-h, 15-kHz pure tone (orange bar) followed by 3 h of silence. Mice were injected with 4-OHT (50 mg/kg) at the midpoint of sound exposure. ***B***, Schematic drawing of the CN in the sagittal plane showing the tonotopic arrangement of AN fibers (colored lines) bifurcating into the aVCN and pVCN. The * indicates the small cap region in B (as well as in ***C1*** and ***D***). ***C1***, Immunohistochemistry staining in a parasagittal VCN slice of 4-OHT-injected TRAP::hVOS mice exposed to sound. Staining for CeFP, the fluorescent moiety of the hVOS probe (Wang et al., 2010), revealed probe expression (red). Tubulin immunostaining highlights AN axons (TuBB3, green). DAPI stains nuclei (blue). hVOS expressing cells distributed along the bands are outlined with a white dashed box in pVCN and aVCN (as well as in ***C2***, ***D***, and ***E1***). The isofrequency band elongated in pVCN ends before entering the octopus cell area (oct). ***C2***, Enlargement of pVCN area from (***C1***); the isofrequency band (outlined) is parallel with the AN fibers labeled with tubulin immunofluorescence (green, arrows). ***D***, hVOS expressing cells in an Isofrequency band in pVCN in a coronal slice. ***E1***, The lower portion of ***C1*** is displayed with only red hVOS staining (no TUBB3 or DAPI) to highlight the isofrequency lamina more clearly (white outlined boxes). ***E2***, The corresponding region as in ***E1*** within a parasagittal VCN slice of a mouse that had undergone the same protocol illustrated in Figure 1*A*, but with no sound. hVOS staining was viewed with twofold higher laser intensity and 70% higher detector sensitivity to bring the brightness in this section to a level comparable to the brightness in ***E1***. The regions containing the isofrequency bands in E1 contained much less labeling. Scale bars = 100 μm (***C1*** and ***D***) and 50 μm (***C2***).

For auditory stimulation, mice at P16–P30 were housed in a custom soundproof box, and received sound stimulation as illustrated in Figure 1*A*. After three quiet hours, continuous sound was delivered for 6 h at ∼90 dB using BioSigRP software (Tucker-Davis Technologies). A 15-kHz tone of 20-ms duration with 2-ms rise and fall time was presented at a rate of 41 Hz (leaving 4.4-ms gaps). The sound was produced by a PC sound card, processed by RZ6 Multi-I/O Processer (Tucker-Davis Technologies), and delivered via a speaker (Ignite) mounted directly above the animals. The sound was paused for no more than 5 min after the first 3 h to administer an intraperitoneal injection of 160 mg/kg Tamoxifen or 50 mg/kg 4-OHT. Tamoxifen was first dissolved in absolute EtOH, then diluted 1/10 by volume with sunflower oil followed by 2-h sonication or overnight nutation, and stored at 4°C in the dark for up to one week. 4-OHT was first dissolved in absolute EtOH at 20 mg/ml, and diluted with Chen oil (a 1:4 mixture of castor oil:sunflower seed oil) to 10 mg/ml for injection (Guenthner et al., 2013). After sound stimulation, mice were kept in quiet for another 3 h before returning to their home cage, and killed 4–5 d later for experiments. This protocol has been shown to produce tonotopic labeling in DCN and VCN from TRAP mice crossed with the Ai14 reporter line (Guenthner et al., 2013). The time line is consistent with the time window determined by the pharmacodynamics of 4-OHT in mice (Robinson et al., 1991).

### Chemicals

Chemicals were from Sigma-Aldrich unless stated otherwise.

### Slice preparation

The brain was removed following decapitation and the dissection performed in artificial CSF (aCSF) at 24–27°C as described previously (Oertel, 1983). aCSF contained (in mm) 130 NaCl, 3 KCl, 1.2 KH_2_PO_4_, 2.4 CaCl_2_, 1.3 MgSO_4_, 20 NaHCO_3_, 6 HEPES, 10 glucose, and 0.4 ascorbic acid, and was saturated with 95% O_2_/5% CO_2_ (pH7.3–7.4). The osmolality was 306 mOsm/kg (3D3 Osmometer; Advanced Instruments). Coronal acute slices of VCN (250–300 μm) were cut with a vibratome (Leica VT 1200).

### hVOS imaging

Slices were superfused with 95% O_2_/5% CO_2_-bubbled aCSF containing 4 μm dipicrylamine (DPA; City Chemical, LLC) and 1 μm strychnine for 30 min before experiments. DPA partitions into the cell membrane and its voltage-induced movement modulates a Förster resonance energy transfer interaction with the cerulean fluorescent protein (CeFP) of the hVOS probe to give a voltage-sensitive fluorescent signal (Chanda et al., 2005; Wang et al., 2010). Bath temperature was measured between the inflow of the chamber and the tissue with a microprobe thermometer. An inline heater warmed the aCSF just before it reached the chamber to keep the temperature between 28°C and 31°C. hVOS imaging was conducted with an Olympus BX51 microscope equipped with a 29-W, 435 nm LED light source (Prizmatix), a standard cyan fluorescent protein filter set, and an Olympus XLUMPlanFl 20× objective (NA 1.0) or LUMPlanFl 60×objective (NA = 0.90). Images were acquired with a CCD-SMQ camera (Redshirt Imaging, now SciMeasure) at 2000 fps with 80 × 80 resolution. A computer program, either Neuroplex (SciMeasure) or an in-house program (Chang, 2006), controlled the timing of illumination, stimulation, and data acquisition. hVOS images were acquired as averages of 5–10 trials at 15-s intervals.

### Voltage-sensitive dye imaging

Slices were stained for 45 min in aCSF that contained 0.05 mg/ml of the voltage-sensitive absorbance dye RH482 (NK3630, Hayashibara Biochemical Laboratories). As with the hVOS experiments, the perfusing aCSF was warmed to maintain a temperature of 28–31°C. Voltage imaging employed a 464-element photodiode fiber optic camera (Wu and Cohen, 1993; Jin et al., 2002; Jackson, 2020). Transmitted light varies with voltage because of the change in dye absorbance. Photocurrent was amplified to 5 V/nA of photocurrent, low-pass filtered at 500 Hz and digitized at 10 kHz. Displayed traces are 20 trial averages. A Prizmatix UHP-white-T-LN LED provided illumination through a 705/30-nm bandpass filter. Light was collected with a Zeiss 10× Plan-Apochromat objective (NA 0.5). Data acquisition, illumination, and stimulation were controlled by in-house software (Chang, 2006).

### Electrical stimulation

To stimulate slices, 50- to 350-μA current pulses were applied with a model A365 stimulus isolator (World Precision Instruments) via aCSF-filled glass electrodes with a ∼10-μm tip opening. The electrode was placed in visually identifiable bundles of AN fibers. Pulse durations were 0.18 ms for hVOS experiments and 0.20 ms for voltage-sensitive dye experiments. For hVOS imaging pulse trains were generated with Clampex 9.2 (Molecular Devices) running on a different computer from that used for imaging, with triggering from the imaging computer initiated by Neuroplex.

### Immunohistochemistry

Mice were first deeply anesthetized with a combination of ketamine (100 mg/kg) and xylazine (10 mg/kg), then transcardially perfused with PBS, followed by ice-cold 4% paraformaldehyde (PFA). After removing the cerebral cortex, the brainstem was left in the skull and postfixed in the same fixative overnight at 4°C. Parasagittal or coronal sections, 40–50 μm thick, of the CN were cut with a vibratome (Leica 1000S).

Free floating sections were collected, permeabilized, blocked with 3% BSA, and incubated with primary anti-GFP antibody (rabbit, Abcam catalog #ab290, 1:500) overnight on a shaker at 4°C to preserve the fluorescent signal of CeFP of the hVOS probe in TRAP-labeled neurons. In some experiments, sections were double stained with anti-tubulin β-III antibody (TUBB3, mouse, BioLegend catalog #801202, 1:500), as a neuronal marker. Sections were then incubated with fluorescent-dye conjugated secondary antibodies for 2 h (Alexa 568 anti-rabbit, Abcam catalog #ab175692, 1:500; Alexa 488 anti-mouse, 1:500, ThermoFisher Scientific catalog #A21202) and counterstained with DAPI for 10 min at room temperature. Images were acquired with a confocal microscope (Nikon A1RS) and processed with ImageJ.

### Data analysis and statistics

For hVOS imaging experiments, regions of interest (ROI) were selected manually with Neuroplex software or with in-house software (Chang, 2006). Response intensity maps were binned at 2× or 4×, depending on the objective used, and a Gaussian spatial filter was applied to aid selection of ROIs. Because depolarization reduces fluorescence in hVOS imaging (Chanda et al., 2005) traces were inverted for display. Fluorescence signals were divided by resting light intensity, and filtered at 500 Hz with a four-pole low-pass Butterworth filter (in Neuroplex), or with a nine-point binomial filter (in-house software). Signals with peaks over three standard deviations above the baseline were included in the analysis. For voltage-sensitive dye imaging, analysis was conducted with the same in-house software used for data acquisition. Amplitude, half-width, and latency (defined as time from stimulus to half peak) were analyzed with Neuroplex. Clampex 9.2, Origin 9 (OriginLab), Igor (WaveMetrics) were used for further analysis and plotting. Statistical tests were performed with Prism (GraphPad Software) and Origin 9.

## Results

### Labeling of isofrequency bands

We first evaluated the distribution of hVOS probe expression in slices of VCN from TRAP::hVOS mice exposed to auditory stimulation with 15-kHz sound (see Materials and Methods), a frequency that mice detect at lowest intensity. With the TRAP strategy, Cre recombinase is expressed from the locus of the immediate early gene *c-fos* in neurons that are active during the time window created by Tamoxifen or 4-OHT injection (Guenthner et al., 2013). In the present study with TRAP::hVOS animals, we employed a 12 h time window (Fig. 1*A*), including 3-h quiet time in sound-proof box, 3-h pure tone stimulation at 15 kHz before 4-OHT injection, 3-h stimulation after injection, and another 3-h quiet time. We found that this protocol drove strong labeling of neurons along the isofrequency band corresponding to 15 kHz, without inducing significant labeling in unrelated isofrequency bands. A similar but shorter time window was adopted in another study using the TRAP approach (Allen et al., 2017).

We visualized expression of the hVOS probe by immunostaining for its fluorescent moiety, CeFP. Figure 1*B* illustrates the relevant CN circuitry and the tonotopic organization of the AN fibers. Immunostaining for tubulin β-III was used as a general neuronal marker and revealed the AN fibers. In sagittal sections, we observed CeFP-expressing neurons distributed in a band that ran parallel to the corresponding tubulin β-III-labeled AN fibers in both pVCN and aVCN (Fig. 1*C2*). The isofrequency CeFP-labeled bands corresponding to 15 kHz were located roughly at the center along the dorsoventral axis, consistent with previous observations of the tonotopic maps in mice (Muniak et al., 2013). Bands tuned to higher frequency occupy more dorsomedial positions through the pVCN and aVCN. The pVCN branch of the band ended before the octopus cell area (oct in Fig. 1*B*,*C1*), located at the most dorsal-caudal tail of the pVCN. Octopus cells were not labeled, presumably because their pure tone thresholds are high (Godfrey et al., 1975; Rhode and Smith, 1986). Also, if they did respond, they would only fire a single onset spike to the stimuli we used (Rhode et al., 1983; Oertel et al., 2000; Smith et al., 2005; McGinley et al., 2012; H.W. Liu et al., 2022), which would presumably be less effective in driving probe expression. The small cell cap, a region that receives both auditory and somatosensory input (Shore and Zhou, 2006), also contained TRAP-labeled neurons (* in Fig. 1*B–D*). In coronal sections, an isofrequency band could be observed in the middle of the dorsoventral axis of this region (Fig. 1*D*), as demonstrated previously in TRAP mice crossed with the Ai14 Cre reporter (Guenthner et al., 2013). The lower portion of the image in Figure 1*C1* is presented with only hVOS staining (red), and without overlaying the tubulin and DAPI fluorescence (Fig. 1*E1*). This makes the isofrequency laminae (white dashed boxes) more clearly visible. The observation of isofrequency bands depended on exposure to sound. Sections were prepared from mice undergoing the protocol in Figure 1*A*, but without sound (Fig. 1*E2*). This image was acquired with twofold higher laser intensity and 70% higher detection sensitivity. Although these settings increased the brightest regions to a level similar to Figure 1*E1*, bands of hVOS-labeled cells were not evident (Fig. 1*E2*).

### hVOS imaging of activity within an isofrequency band

**Figure 2.**
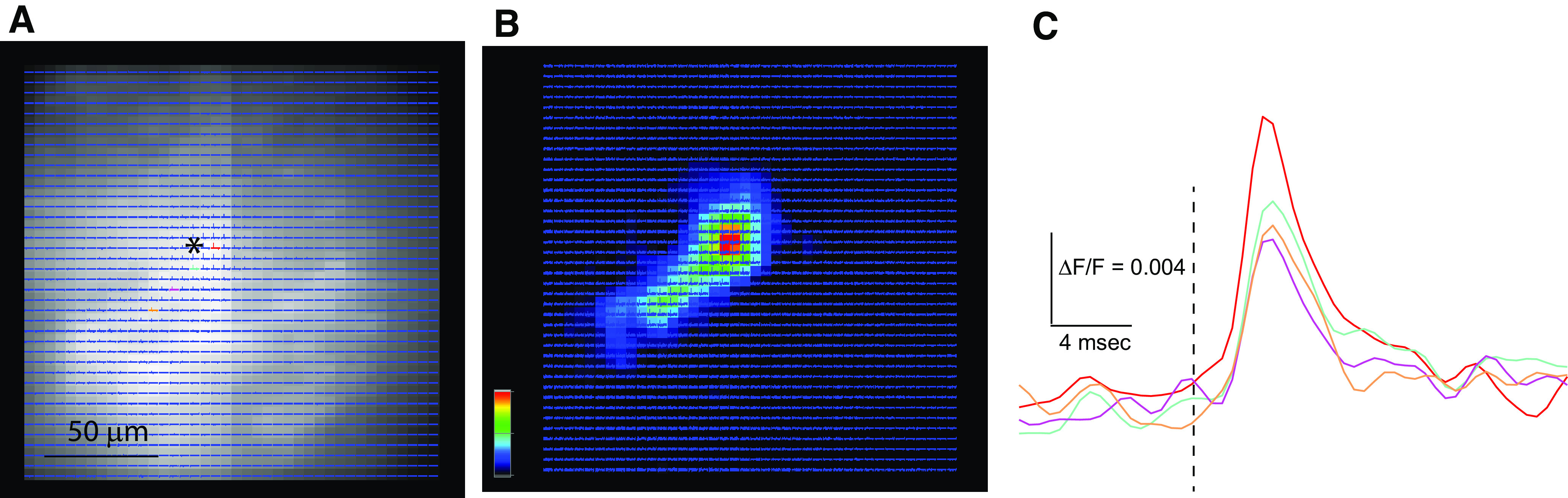
hVOS imaging of responses to a single pulse in a coronal VCN slice from a TRAP::hVOS mouse. ***A***, A resting fluorescence image with overlain traces binned 4:1 (traces on expanded scales are shown in ***C***). The site of AN stimulation is labeled with *. Selected traces are highlighted with colors for display in ***A***. ***B***, Peak ΔF/F encoded as color following the scale in the lower left corner, normalized to the maximum signal in the field of view. The band of green extending diagonally from the site of stimulation is suggestive of an isofrequency band. The colored scale to the left indicates normalized depolarization. ***C***, Four selected traces indicated by color in A illustrate the response with higher amplitudes near the site of stimulation (red). The dashed line marks the time of stimulation. Traces are 10 trial averages.

Having validated probe targeting, we then used fluorescence imaging in brain slices to evaluate the responsiveness of labeled neurons in the VCN of TRAP::hVOS mice. We focused specifically on the activity of neurons such as those displayed in Figure 1, which had been electrically active when the mouse was exposed to 15-kHz pure tone stimulation. In a VCN slice from a TRAP::hVOS animal, we placed a stimulating electrode in the AN fibers (indicated in a resting fluorescence image, * in Fig. 2*A*), applied a single pulse, and imaged fluorescence. Voltage changes move DPA and change its distance to the fluorescent protein, altering their Förster resonance energy transfer and changing the fluorescence emission. Fluorescence thus tracks voltage changes during synaptic and action potentials, as demonstrated by simultaneous hVOS imaging and patch clamp recording in cultured neurons (Wang et al., 2010) and brain slices (Ghitani et al., 2015; Bayguinov et al., 2017). Fluorescence traces are overlain to illustrate the spatial resolution of imaging, but with all traces thus displayed individual traces are too small to judge. The spatial distribution of responses can be assessed by encoding the maximum stimulus-induced fluorescence change, ΔF/F, at each location as color, and mapping. This map shows that the most responsive locations fall along a distinct band (Fig. 2*B*, green-to-red). The band of labeled cells is not evident in the resting fluorescence image because of autofluorescence (Fig. 2*A*). Figure 2*B* shows that responses are greater in amplitude close to the site of stimulation but are clear over distances of >50 μm. Selected fluorescence traces from the locations highlighted with color in Figure 2*A* are displayed in Figure 2*C* to show the time course of voltage changes more clearly at specific locations. These traces indicate that peak amplitudes range from ∼0.01 near the stimulus electrode (red trace) to ∼40% lower (0.006%) at more distant sites (colors corresponding to traces in Fig. 2*A*). Response half-widths were close to 4 ms. Response latencies following stimulation were ∼1.5 ms, which is comparable to the synaptic delays of shock-induced AN inputs to CN cells measured electrophysiologically in slices (Ferragamo et al., 1998; Cao and Oertel, 2010).

### Voltage-sensitive dye imaging of population activity

**Figure 3.**
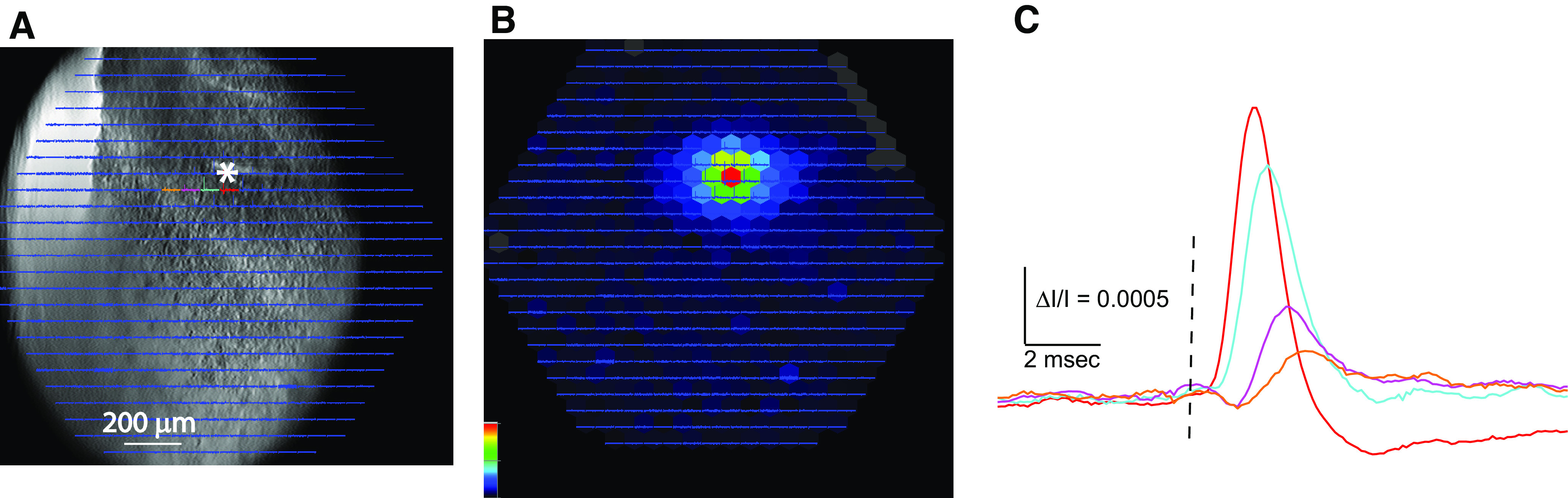
Voltage-sensitive dye imaging of responses to a single pulse in a coronal VCN slice from a non-hVOS mouse not subjected to the sound protocol. ***A***, A CCD image of the slice with traces overlain. The site of stimulation is labeled with * and selected traces highlighted with colors for display in ***C***. ***B***, Peak ΔI/I encoded as color following the scale in the lower right corner, normalized to the maximum. Red indicates largest depolarization. Response amplitudes decline radially from the site of stimulation. ***C***, Traces selected from ***A***, ***B*** illustrate the form of the responses, with amplitude decreasing and latency increasing with distance from the site of stimulation. Traces are 20 trial averages.

To compare the overall electrical responses to single pulses with that just described in an isofrequency band in hVOS imaging (Fig. 2), we stained VCN slices with RH482, a voltage-sensitive absorbance dye that stains membranes nonselectively. This sensor operates through an electrochromic mechanism in which the electric field across the membrane shifts the absorbance spectrum (Momose-Sato et al., 1999; Loew, 2015). By illuminating with a frequency band above the absorbance maximum we can image voltage as electrically-evoked changes in transmitted light (Fig. 3). A static transmitted light image of the slice is shown in Figure 3*A* with overlain optical traces. Single pulse stimulation elicited absorbance changes that spread radially rather than within a band as seen in the hVOS slices. Again, the many traces in the overlay (Fig. 3*A*) are too small to assess. Mapping maximal stimulus-evoked light changes, ΔI/I, encoded as color illustrate the spatial distribution of voltage spread (Fig. 3*B*). Note that these images were acquired at a sixfold lower magnification compared with the hVOS experiments of Figure 2. In these voltage-sensitive dye experiments, we could see clear responses at distances of ∼250 μm that revealed a roughly radial spread (Fig. 3*B*), compared with the band seen with hVOS (Fig. 2*B*). This difference is consistent with the view that the activity seen in the hVOS experiment represents an isofrequency band while voltage-sensitive dye imaging reveals responses that are not restricted to an isofrequency band.

Traces were selected to show the responses in greater detail on an expanded scale (Fig. 3*C*; colors corresponding to colored traces in Fig. 3*A*). Response amplitudes decline in a graded fashion with distance from the site of stimulation because at greater distances the activated fibers and cells are sparser. In contrast to the nearly 4-ms half-widths of the hVOS signals (Fig. 2*C*), the voltage-sensitive dye signal half-widths are roughly 1.5 ms (Fig. 3*C*) as expected for action potentials. The longer half-widths of the hVOS responses indicate that they have contributions of synaptic potentials from cell bodies and dendrites. The traces in Figure 3*C* show a clear increase in latency with distance from the site of stimulation. The slope of latency-distance plots was used to determine a conduction velocity of 0.273 ± 0.036 m/s (mean ± SE, *N* = 8).

### Excitatory synaptic receptor dependence

**Figure 4.**
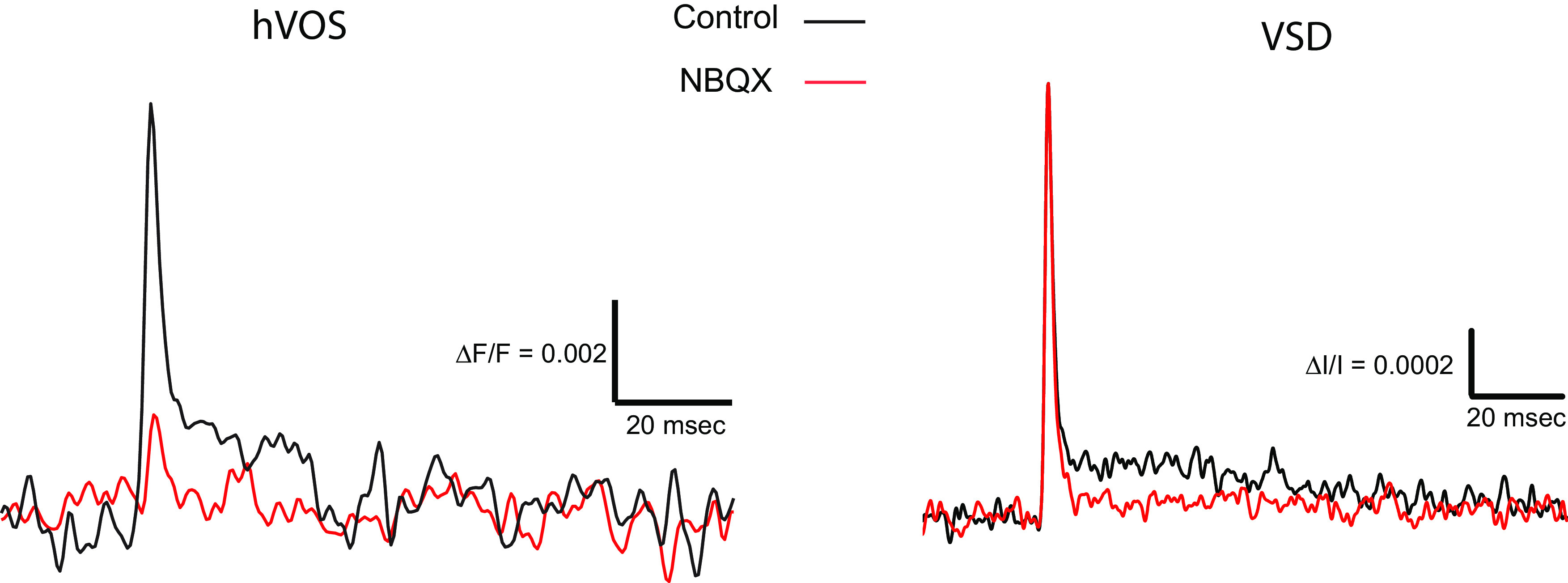
NBQX action on hVOS and voltage-sensitive dye (VSD) responses. A single pulse response was recorded before (Control, black) and ∼20 min after (NBQX, red) application of 10 μm NBQX. NBQX blocked most of the hVOS response. In the voltage-sensitive dye response NBQX had no effect on the initial spike but blocked the later synaptic component. Traces are 10 trial averages for both VSD and hVOS.

To explore the different components of VCN circuitry probed by hVOS and voltage-sensitive dye imaging, we tested the action of glutamate receptor antagonists, which have proven useful in identifying excitatory synaptic contributions to both voltage-sensitive dye (Kojima et al., 1999; Tsutsui et al., 2001) and hVOS (Ma et al., 2021) signals. Here, we found that the antagonist 2,3-dioxo-6-nitro-1,2,3,4-tetrahydrobenzo-benzo(f)quinoxaline (NBQX; 10 μm) blocked the fluorescence changes in hVOS imaging of TRAP-labeled cells elicited by AN fiber stimulation (Fig. 4*A*). The small residual signal could reflect an incomplete block of synaptic responses in VCN neurons, or a small amount of direct depolarization by the stimulating electrode. It is also possible that some hVOS probe is expressed in the axons of AN fibers since the cell bodies of origin of these axons also experience electrical activity during sound stimulation, and voltage changes in their axons would be insensitive to NBQX. The strong blockade illustrated in Figure 4*A* indicates that the hVOS signals represent predominantly postsynaptic responses to glutamate release from AN fibers.

We then tested the action of NBQX on voltage-sensitive dye signals. NBQX did not reduce their peak amplitude, indicating that these voltage changes are generated predominantly by axonal/presynaptic AN action potentials. However, traces taken at a distance from the site of stimulation often have a small depolarization after the spike, and this late component was blocked by NBQX (Fig. 4*B*, red trace). Thus, the voltage-sensitive dye signals reflect predominantly presynaptic action potentials, with a small contribution from excitatory postsynaptic potentials. The different action of NBQX in hVOS and voltage-sensitive dye experiments supports the distinctive nature of the circuit elements probed with these two imaging techniques.

### hVOS imaging with train stimulation

**Figure 5.**
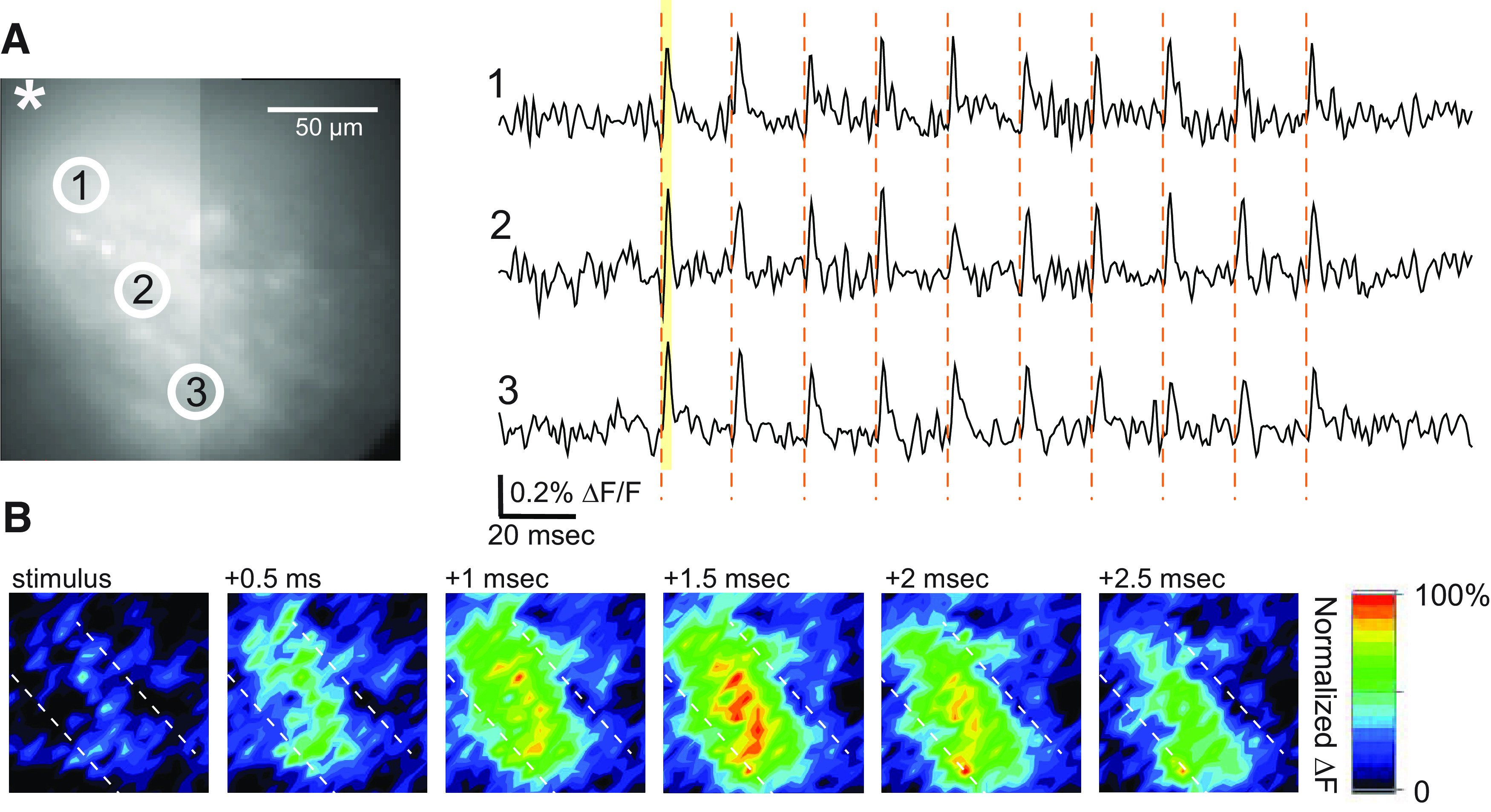
hVOS imaging of responses to train stimulation in a VCN slice from a TRAP::hVOS mouse. Labeled cells were depolarized by stimulus trains applied to the AN. ***A***, Left, An epifluorescence image showing the stimulus site (*) and numbered regions of interest selected for trace display (circles). Right. Fluorescence traces from three locations indicated in the image along the isofrequency band in VCN. A 10-pulse train at 50 Hz (300 μA, times of pulses indicated by vertical red dashed lines) elicited depolarizing responses. Traces are 10 trial averages. ***B***, A sequence of response intensity snapshots at the indicated times (0.5-ms intervals) after the first pulse. The yellow shaded region in the right panel in ***A*** highlights the time interval used to generate the maps. After stimulation, responses appear uniformly along the isofrequency band (boundaries indicated by white dashed lines). The colored scale to the right indicates depolarization as red, normalized to the maximum signal in the field of view. The maximal response is reached in the 1.5-ms map.

**Figure 6.**
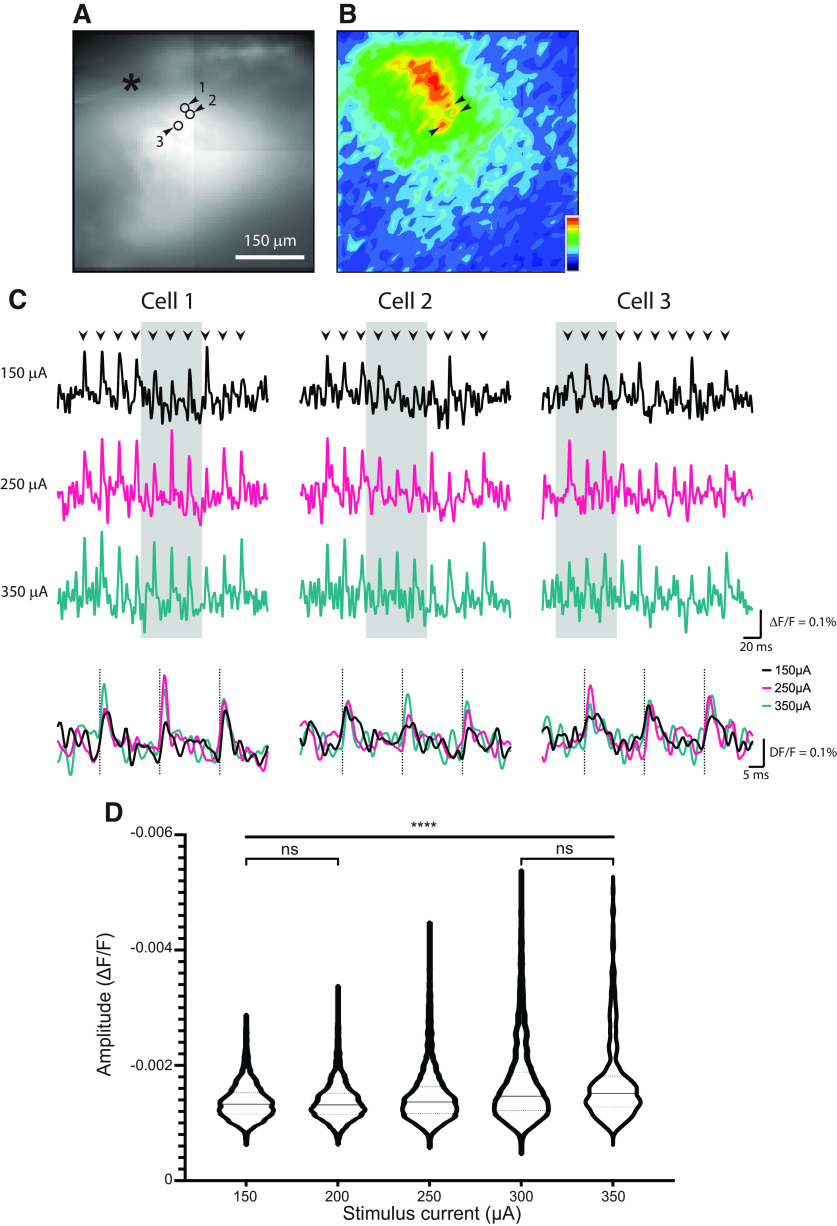
hVOS imaging response amplitudes and train stimulation. ***A***, Epifluorescence image of the VCN slice showing the stimulus site (*), and three small circles and arrowheads indicating sites selected for display of traces. Traces are 10 trial averages. ***B***, Map of peak ΔF/F during a 50 Hz train of 10 pulses (250 μA). The responsive region forms a band corresponding to an isofrequency lamina (note the lower magnification compared with Fig. 5). ***C***, Responses of the three locations highlighted in ***A*** and ***B***, to 150 μA (black), 250 μA (red), and 350 μA (blue). Traces in the gray shaded area are expanded and overlaid and stimulation times indicated with gray dashed lines. ***D***, Response amplitude versus stimulation current in violin plot format. Amplitudes from slices from five animals with 2–128 locations evaluated per experiment. Differences were significant across five stimulation currents in pairwise comparisons, except for the first two and the last two groups (****, Kruskal–Wallis test with Dunn’s test, *p* < 0.0001). The results suggested that low stimulus currents evoke predominantly synaptic potentials and that higher stimulus currents evoke action potentials in a small number of cells.

Because sounds can generate trains of spike activity in AN fibers, we applied trains of 10 pulses at 50 Hz to AN fibers and recorded hVOS signals in VCN slices from TRAP::hVOS mice exposed to 15-kHz sound. We observed fluorescence changes that were nearly synchronous throughout an isofrequency band (Fig. 5*A*), and ΔF/F response maps at half millisecond intervals revealed the synchronous activation throughout the band (Fig. 5*B*). Trains of stimulation were applied to the slice seen in a resting light image in Figure 6*A*, and Figure 6*B* displays the peak ΔF/F for the entire stimulus window (rather than a sequence of snapshots at 0.5-ms intervals as in Fig. 5*B*). The lower magnification used in this experiment (20× objective instead of 60×) makes visible the extent of the isofrequency lamina (Fig. 6*B*).

To assess the contributions of action potentials and synaptic potentials we varied the stimulus current of pulses applied in trains. The responses from three representative locations to stimulation with 150, 250, and 350 μA are compared in Figure 6*C*. We then evaluated signal amplitudes across experiments. Violin plots indicated a significant increase in response amplitude with current (Fig. 6*D*). Pairwise comparison with Dunn’s test showed that responses to the two strongest stimulations (300 and 350 μA) were significantly greater than to the two weakest (150 and 200 μA). The events in the low stimulation groups with an average ΔF/F around 0.0013 were likely to be subthreshold. With higher stimulation current we observed an increased proportion of responses with ΔF/F above 0.003. These are likely to be suprathreshold, and are comparable in amplitude to action potentials seen in hVOS signals from a variety of other cell types in slices from other brain regions (Ghitani et al., 2015; Bayguinov et al., 2017).

## Discussion

Here, we used two different voltage imaging techniques to reveal circuit activity in the first station of the auditory pathway in mouse brain slices. Two very different optical probes were used in the VCN to transduce membrane potential into optical signals. The voltage-sensitive dye (RH482) is a synthetic organic molecule that stains neurons and their processes nonselectively to provide a more global picture. Although the dye stains membranes nonselectively, the primary source of stimulus-evoked voltage changes viewed with this probe appears to be axons. This assignment is consistent with the brief half-width of the recorded events, their insensitivity to the glutamate receptor antagonist NBQX, and their rapid spread. The postsynaptic contribution to these signals is slow and relatively small. By contrast, hVOS imaging depends on a genetically-encoded component. This enabled us to use an activity-based probe targeting strategy to focus on a unique population of neurons within the VCN that are activated by 15-kHz sound stimulation. The longer half-widths and NBQX sensitivity of these signals indicate they are postsynaptic. These two different voltage imaging techniques thus targeted distinct neuronal populations and allowed us to study two elements of VCN circuitry with complementary functions.

The axonal targeting of voltage-sensitive dye enabled us to determine a conduction velocity of 0.27 m/s. This is on the high end within the range of unmyelinated axonal conduction velocities in various CNS preparations (Eccles et al., 1967; Ma et al., 2017; Jackson, 2020; Scheuer et al., 2022) and well above values in nonmammalian systems (Senseman et al., 1983; Konnerth et al., 1987). Our value is quite similar to a value of 0.3 m/s, measured from the unmyelinated parallel fiber pathway formed by granule cell axons in the superficial layer of the guinea pig DCN (Manis, 1989). Conduction is much faster in myelinated Type I AN fibers: 22 m/s in humans (Møller et al., 1994) and 2.7 m/s in rats (Tagoe et al., 2014). Thus, the voltage-sensitive dye probably preferentially reports signals from unmyelinated Type II AN fibers. Factors that could account for targeting these fibers include greater dye access to unmyelinated fibers and the small area of node membrane. Very little is known about the function of Type II AN fibers because their small size makes them very difficult to study with conventional recording methods. Some evidence indicates they may act as nociceptive monitors of the cochlea (C. Liu et al., 2015). The somewhat higher velocity compared with many other conduction velocities of unmyelinated axons noted above may reflect adaptations in VCN for more rapid computations. Voltage-sensitive dye imaging may provide a powerful method for further study of Type II AN fiber activity.

In contrast to voltage-sensitive dye imaging, the TRAP strategy (Guenthner et al., 2013) enabled us to target the genetically-encoded hVOS probe to a specific set of neurons defined by their function. By using offspring from crosses of the TRAP mouse with our hVOS Cre reporter (Bayguinov et al., 2017), we could drive probe expression selectively in a distinct population of CN neurons defined by their function. hVOS responses of these neurons showed a laminar spread (Figs. 2, 5, and 6) that is distinct from the roughly circular spread of voltage-sensitive dye responses (Fig. 3). Thus, this TRAP-hVOS imaging strategy reveals the tonotopic organization of the CN (Luo et al., 2009; Muniak and Ryugo, 2014), and indicates that we were observing responses of neurons with a CF of 15 kHz. Previous physiological studies of CN neurons tuned to the same frequency have only been performed *in vivo* on a limited number of neurons over multiple experiments (Blackburn and Sachs, 1989, 1990; Prell et al., 1996; May et al., 1998). Efforts to focus on cells in a slice *in vitro* with a given CF have had to rely on the general location within the slice. The present approach with TRAP-based targeting of a genetically-encoded voltage sensor defined these cells directly within slices under study, and allowed us to record *in vitro* simultaneously from a population of neurons with the same CF. TRAP-targeting with sound should be generalizable beyond the VCN, and provide an approach to the study of neural circuitry in other parts of the auditory brain stem with tonotopic organization such as the DCN, medial and lateral superior olive, and medial nucleus of the trapezoid body (Kandler et al., 2009). Using this approach to characterize sound-responsive neurons in regions without tonotopic organization could also be interesting.

We observed rapid synchronous activation of the neurons within an isofrequency band in response to AN stimulation. This was made possible by the rapid response dynamics of hVOS imaging, which reflects the <0.5-ms transit time for DPA to cross the membrane, at both physiological and room temperature (Chanda et al., 2005; Bradley et al., 2009; Wang et al., 2010). As a result, hVOS imaging can track action potentials with high temporal fidelity (Ghitani et al., 2015; Ma et al., 2019) and recapitulate action potential half-widths of distinct cell types (Bayguinov et al., 2017). hVOS imaging tracks the activity of many neurons within a field of view. Thus, with high temporal resolution simultaneous recordings from multiple neurons, hVOS has the potential to reveal important elements of auditory computation. Although our targeting strategy was not based on cell type but on physiology, it is likely that we were looking at bushy cells or T-stellate cells or both (Cao and Oertel, 2010). Because of the different projection targets and molecular expression patterns of VCN neurons, it should be possible to combine the strategy employed here with retrograde tracing or other Cre drivers to study the circuitry of specific cell types (Pór et al., 2005; Ngodup et al., 2020).

While our hVOS response maps revealed some small hotspots with higher peak ΔF/F (Figs. 5*B*, 6*B*), at this stage we cannot determine whether these hotspots represent individual cells or multiple closely aligned cells. In other brain regions hotspots in hVOS ΔF/F maps align well with cells in simultaneous patch clamp recordings (Ghitani et al., 2015; Bayguinov et al., 2017), as well as with cells visible in fluorescence images (Bayguinov et al., 2017; Ma et al., 2021). Furthermore, cluster analysis methods have proven effective in identifying responses arising from single parvalbumin interneurons in cortical slices, even when the cells are not visible in fluorescence images (Canales et al., 2022; Scheuer et al., 2022). It should be possible to reach this level of resolution with hVOS in VCN slices, especially if cells can be labeled more sparsely. A weaker sound stimulus might achieve sparser labeling, and sound protocols that target different populations might give us cell-type specificity. As an example, octopus cells (Fig. 1) only detect (fire in response to) coincident firing of large groups of AN fibers, while the pure tone used here is a very poor stimulus for these cells (Ferragamo and Oertel, 2002; Smith et al., 2005; H.W. Liu et al., 2022). In contrast, octopus cells respond very robustly and accurately to click stimuli at high rates while other VCN neurons do not. This stimulus could thus label octopus selectively. Cre drivers can label specific cell types in the CN in a manner that is complementary to the present approach based on sound stimulation. With the extraordinary flexibility of genetically-encoded voltage sensor targeting, the present study creates a wealth of opportunities for the study of the sound processing circuitry of the CN.

## Synthesis

Reviewing Editor: Miriam Goodman, Stanford University Decisions are customarily a result of the Reviewing Editor and the peer reviewers coming together and discussing their recommendations until a consensus is reached. When revisions are invited, a fact-based synthesis statement explaining their decision and outlining what is needed to prepare a revision will be listed below. The following reviewer(s) agreed to reveal their identity: Michael Lin, Aditya Singh.

In your revision, please strengthen the case for voltage imaging in general and in the context of the use of TRAP-labeling in this system. Please also conduct additional control experiments suggested by Rev1. These were considered important experiments to demonstrate the specificity of TRAP labeling by both reviewers in post-review discussions. The revision should incorporate the results of these validation/control experiments regarding interpretation of the resulting signals.

Rev1

n this work, the authors perform TRAP to express the genetically targeted sensor hVOS in neurons in the ventral cochlear nucleus (VCN) that respond to a particular sound frequency. Then, in VCN slices from these animals, they examine electrode-induced voltage changes in the TRAPed cels using hVOS. They also examine electrode-induced voltage changes using RH482, a dye whose absorbance changes with voltage. Both indicators are visualized with or without GluA blockade by NBQX, and hVOS is also visualized with trains of electrical stimulation at various intensities. The authors conclude hVOS responses report postsynaptic potentials while RH482 mostly reports axonal action potentials.

The study is interesting and the interpretations may well be supported by the data, but there is some uncertainty that can hopefully be resolved quickly.

First, it is not clear how specific the TRAP technique is. In Fig 1, a different frequency should be used to show there is specificity, as well as a no-sound control. If the former is not available, perhaps it can be omitted, but the latter is certainly important.

Second, it is unclear where the electrode is relative to axons going to the TRAPed cells, and the TRAPed cell bodies. If the AN electrode stimulus is far from the band of TRAPed cells in the VCN, then why does the claimed postsynaptic hVOS response surrounds the stimulus? If close, then the residual hVOS response to electrical stimulation after NBQX could be direct depolarization of the postsynaptic TRAPed hVOS+ cells by the stimulus, but the authors don’t bring up this possibility.

Minor:

1. The individual fluorescence traces are hard to see in Fig 2B and 3A. Suggest using green.

2. Fig 6 legend should mention hVOS.

3. Legends for hVOS figures should mention that traces are averages of 5-10 as this would explain the smoothing of the noise.

4. The authors should explicitly discuss published data on the kinetics of hVOS at the appropriate temperature to ensure that the longer delay to peak of hVOS signals vs RH482 is not simply due to slower kinetics.

Rev2

In the study titled, “Imaging Voltage Globally and in Isofrequency Lamina in Slices of Mouse Ventral Cochlear Nucleus (CN)” authors take an ambitious approach with a TRAP:hVOS Tg mouse line where tamoxifen induced cFos-coupled Cre-expression allows hVOS to be expressed and embedded in the inner membrane of 15kHz-pure-tone sensitive neurons in CN. For the first time in the field, this potentially allows in vitro access to the same 15kHz-pure-tone sensitive neurons that were tagged with hVOS in vivo. Authors obtain CN slices from the same mice and characterize the neural activity within isofrequency lamina in response to the auditory nerve fiber stimulation using voltage-sensitive optical imaging. Authors also use a second approach with voltage sensitive dye to characterize electrical stimulation induced neuronal response in the CN slices that do not have hVOS tagged neurons. Interestingly, voltage-sensitive dye reveals radially distributed response as compared to the isofrequency band response observed in hVOS slices. Overall, the manuscript is very well written. Although, the approach for genetic targeting isofrequency lamina neurons does require some clarifications as listed below along with other concerns.

Introduction and Discussion -

- Well written, no concerns.

Material and Methods -

- For voltage sensitive dye-based imaging experiments, did authors try to test CN slices from TRAP-hVOS mice without tamoxifen induction or extended 15kHz pure-tone exposure? What was the rationale behind using other mice for voltage sensitive dye imaging?

- How long is the duration for which intraperitoneally injected tamoxifen allows cFos-coupled expression of Cre-recombinase in cortical neurons? Since Cre-recombinase is expressed in all the neurons affected by tamoxifen, hVOS expression is expected to be non-specific. Potential limitations of this approach should be added in discussion.

- What was the rationale for keeping mice alive for 4-5 days after the day of 15kHz pure-tone induced hVOS tagging of neurons?

Did authors try any other frequency than 15kHz to ensure the robustness of this novel technique? Why not?

Results

- It is stated that neurons in a band that ran parallel to the corresponding tubulin beta II labeled AN fibers were likely activated by sound stimulation. The connection between fiber staining and sound activated neurons is not very clear and should be clarified.

- Fiber staining is not clear in Fig 1C1, so authors should refer to 1C2 instead.

- Small cap region indicated by asterisk is not mentioned in the corresponding figure legend, should be added.

- Add description for dashed box in Figure 1D legend

- If possible, Fig 2A, 5A, and 6A should be provided with higher resolution.

## Author Response

Synthesis statement.

In your revision, please strengthen the case for voltage imaging in general and in the context of the use of TRAP-labeling in this system.

To strengthen the case in terms of motivation for using these techniques, we added text to the Introduction (P 5-6). To strengthen the case in terms of the validity of these techniques we added text to the Results, where we cite work validating hVOS with simultaneous patch clamp recordings (P 14), validation of voltage sensitive dyes with wavelength dependence of the charge-shift mechanism (P 15), and prior use of glutamate receptor antagonists to resolve synaptic contributions in voltage imaging experiments (P 16).

Please also conduct additional control experiments suggested by Rev1. These were considered important experiments to demonstrate the specificity of TRAP labeling by both reviewers in post-review discussions. The revision should incorporate the results of these validation/control experiments regarding interpretation of the resulting signals.

A different frequency would be very valuable but we anticipate a significant challenge in comparing isofrequency lamina in different planes of slicing. However, we had done some no-sound controls at the inception of this study that we neglected to include. We added two new panels to Fig. 1, E1 and E2. Fig. 1E1 presents an expanded view of the image in Fig. 1C1 with only hVOS probe staining to highlight the isofrequency lamina more clearly. New Fig. 1E2 is an image of this region in a slice from a no-sound control mouse. Because with no-sound there was much lower fluorescence in the hVOS channel this image was acquired with 2x laser intensity and 70% higher detector sensitivity. This shows that the isofrequency lamina are absent. There is very little fluorescence in 1E2 in the regions corresponding to the isofrequency lamina in 1E1, and the much lower levels of hVOS probe throughout are only visible because of the higher acquisition settings. Text was added on P 13 to discuss these results. The legend of Fig 1 was extended.

Rev1

First, it is not clear how specific the TRAP technique is. In Fig 1, a different frequency should be used to show there is specificity, as well as a no-sound control. If the former is not available, perhaps it can be omitted, but the latter is certainly important.

The added control experiment was discussed above (Fig. 1E1 and 1E2).

Second, it is unclear where the electrode is relative to axons going to the TRAPed cells, and the TRAPed cell bodies. If the AN electrode stimulus is far from the band of TRAPed cells in the VCN, then why does the claimed postsynaptic hVOS response surrounds the stimulus? If close, then the residual hVOS response to electrical stimulation after NBQX could be direct depolarization of the postsynaptic TRAPed hVOS+ cells by the stimulus, but the authors don’t bring up this possibility.

We added to the Methods that we placed the electrode in visually identifiable bundles of AN fibers (P 10). Regarding he NBQX insensitive component, the residual hVOS response is quite small (red trace in Fig. 4 - left) and we agree that a direct depolarization cannot be ruled out. We incorporated this into our summary of possible explanations in Results on P 16.

Minor:

1. The individual fluorescence traces are hard to see in Fig 2B and 3A. Suggest using green. Unfortunately the software we use to display this data does not offer the option of changing the color of the traces in the overlays. The problem is not just color but also size of display for each trace in arrays that display 1600, and 466 traces. That is why these figures include displays of 4 selected traces to the right (Fig 2C and 3C).

2. A mention of hVOS has been added to the legend of Fig. 6 as requested.

3. The number of trials averages has been added to the figure legends.

4. The authors should explicitly discuss published data on the kinetics of hVOS at the appropriate temperature to ensure that the longer delay to peak of hVOS signals vs RH482 is not simply due to slower kinetics. On P. 21 in the Discussion, we had previously discussed hVOS kinetics and cited the relevant papers (Chanda et al., 2005; Bradley et al., 2009; and Wang et al., 2010). To this discussion we added “at both physiological and room temperature”. In studies at physiological and room temperatures hVOS kinetics is fast enough to track action potentials.

Rev2

Materials and Methods

- For voltage sensitive dye-based imaging experiments, did authors try to test CN slices from TRAP-hVOS mice without tamoxifen induction or extended 15kHz pure-tone exposure? What was the rationale behind using other mice for voltage sensitive dye imaging?

No voltage sensitive dye experiments were performed in TRAP-hVOS mice. The no-sound control that we added (Fig. 1E2) indicated no labeling of isofrequency lamina in the absence of sound. Regarding mouse strains, we used CBA mice for the voltage-sensitive dye experiment because of a long-standing practice of researchers of auditory electrophysiology, including our own lab. A study has shown that hearing is similar in CBA and C57 mice (Walton et al., 1995), and we now mention that in Methods (P 7). We had noted in the original submission that no differences were observed between strains.

- How long is the duration for which intraperitoneally injected tamoxifen allows cFos-coupled expression of Cre-recombinase in cortical neurons? Since Cre-recombinase is expressed in all the neurons affected by tamoxifen, hVOS expression is expected to be non-specific. Potential limitations of this approach should be added in discussion.

The duration of action of tamoxifen (4-OHT) depends on its pharmacodynamics and pharmacokinetics (Robinson et al., 1991). Protocols must be developed empirically for each behavior, and this was done previously by the creators of the TRAP method (Guenthner et al, 2013). We added the following text on P 8: “This protocol has been shown to produce tonotopic labeling in DCN and VCN from TRAP mice crossed with the Ai14 Cre reporter line (Guenthner et al, 2013). The time-line is consistent with the time window determined by the pharmacodynamics of 4-OHT in mice (Robinson et al., 1991)”. Our no-sound control indicates that Cre-recombinase is not expressed in all neurons affected by 4-OHT but only those activated by sound (Fig. 1E2 and P 13).

- What was the rationale for keeping mice alive for 4-5 days after the day of 15kHz pure-tone induced hVOS tagging of neurons?

This is a continuation of the above question. The number of days after sound stimulation reflects the time needed for the initiation of Cre recombinase expression, Cre-induced recombination, and subsequent probe expression. As with the preceding point, this has been determined empirically and we were fortunate that the creators of the TRAP method (Guenthner et al., 2013) worked out the protocol for sound-induced labeling in the cochlear nuclei.

Did authors try any other frequency than 15kHz to ensure the robustness of this novel technique? Why not?

We noted above that testing different frequencies will be challenging. The best we can do at this point is a no-sound control (Fig. 1E2).

Results

- It is stated that neurons in a band that ran parallel to the corresponding tubulin beta II labeled AN fibers were likely activated by sound stimulation. The connection between fiber staining and sound activated neurons is not very clear and should be clarified.

In our discussion of the tubulin staining we now realize there was no need to state “suggesting that these neurons were activated in the mouse by sound stimulation”, so this text was deleted (P 12).

- Fiber staining is not clear in Fig 1C1, so authors should refer to 1C2 instead.

Changed as suggested.

- Small cap region indicated by asterisk is not mentioned in the corresponding figure legend, should be added.

We added a mention of the small cap region to the legend of Fig. 1.

- Add description for dashed box in Figure 1D legend

The figure legend now states that the white dashed boxes highlight bands of probe expressing cells Fig. 1C1, 1C2, D, and 1E1.

- If possible, Fig 2A, 5A, and 6A should be provided with higher resolution.

These are resting fluorescence images taken with the CCD-SMQ camera used for hVOS imaging. Its resolution is 80x80 (mentioned in Methods P 9). A higher resolution image can be taken with another camera coupled to a different camera port, but this image would lack the perfect alignment and focal plane with the color maps of activity.

We hope that these revisions have addressed the reviewers’ and editor’s concerns and that the paper is now acceptable.
